# Cerebral Contribution to the Execution, But Not Recalibration, of Motor Commands in a Novel Walking Environment

**DOI:** 10.1523/ENEURO.0493-19.2020

**Published:** 2020-02-18

**Authors:** Digna de Kam, Pablo A. Iturralde, Gelsy Torres-Oviedo

**Affiliations:** 1 Swanson School of Engineering, Department of Bioengineering, University of Pittsburgh, Pittsburgh, Pennsylvania 15213; 2 Department of Rehabilitation, Donders Institute for Brain, Cognition and Behaviour, Radboud University Medical Center, Nijmegen, HB 6500, The Netherlands; 3 Depto. de Ingeniería, Facultad de Ingeniería y Tecnologías, Universidad Católica del Uruguay, CP 11600, Montevideo, Uruguay

**Keywords:** electromyography, feedback, sensorimotor adaptation, split-belt, stroke

## Abstract

Human movements are flexible as they continuously adapt to changes in the environment. The recalibration of corrective responses to sustained perturbations (e.g., constant force) altering one’s movement contributes to this flexibility. We asked whether the recalibration of corrective actions involve cerebral structures using stroke as a disease model. We characterized changes in muscle activity in stroke survivors and control subjects before, during, and after walking on a split-belt treadmill moving the legs at different speeds. The recalibration of corrective muscle activity was comparable between stroke survivors and control subjects, which was unexpected given the known deficits in feedback responses poststroke. Also, the intact recalibration in stroke survivors contrasted their limited ability to adjust their muscle activity during steady-state split-belt walking. Our results suggest that the recalibration and execution of motor commands are partially dissociable: cerebral lesions interfere with the execution, but not the recalibration, of motor commands on novel movement demands.

## Significance Statement

Corrective responses mediated by feedback have been shown to adapt according to task demands. They also reflect updates in the recalibration of the motor system to sustained and predictable changes in the environment. The extent of cortical involvement in this process is unknown. Here we demonstrate that cortical lesions from stroke alter the execution of motor patterns, but not their recalibration. This is important since it suggests that stroke survivors retain the potential to correct movements through error-based protocols, which is an ability that could be exploited for rehabilitation purposes.

## Introduction

Humans continuously adapt their movements to changes in the body or environment through corrective responses and adjustment of planned actions. Corrective responses are rapidly triggered on unexpected movement disturbances (Jordan and Rumelhart, 1992; Bhushan and Shadmehr, 1999). Conversely, planned actions are predictive in nature and are updated through sustained perturbations (e.g., constant force) altering one’s movement (Wolpert et al., 1998). Recent work has shown that corrective motor commands also adapt to persistent changes in the environment, such that the subjects perceive the novel situation as the new “normal” (Iturralde and Torres-Oviedo, 2019). However, little is known about the neural processes contributing to the recalibration of corrective responses.

It has been suggested that planned and corrective actions share an internal representation of the environmental dynamics (Wagner and Smith, 2008; Maeda et al., 2018); thus, their recalibration could rely on updates to these internal models (Wolpert et al., 1998). If so, the recalibration of corrective responses after sustained exposure to a novel environment is likely dependent on cerebellar structures (Smith and Shadmehr, 2005; Morton and Bastian, 2006), but not on cerebral structures (Reisman et al., 2007; Choi et al., 2009). On the other hand, corrective responses are cerebral dependent, as evidenced by the deficient nature of corrective responses in stroke survivors (i.e., poor muscle coordination, amplitude, or latency; Marigold and Eng, 2006; De Kam et al., 2017, 2018) and impaired task-dependent modulation of this corrective activity (Trumbower et al., 2013; De Kam et al., 2018). Thus, it is plausible that the recalibration of corrective responses is also affected after cerebral lesions, which would imply cerebral-dependent adaptation of corrective actions. Here, we evaluate the involvement of cerebral structures in the recalibration of reactive control through the analysis of corrective muscle activity in individuals with cerebral lesions after stroke.

We characterized stroke-related deficits in muscle activity before, during, and after split-belt walking, which induces robust locomotor adaptation (Reisman et al., 2007). We hypothesized that the execution of motor patterns in a novel walking situation, and the subsequent recalibration of corrective responses would be impaired poststroke. This was based on literature indicating cerebral-related deficits in the modulation of corrective responses (De Kam et al., 2018) and poor muscle coordination poststroke in general (Bowden et al., 2010; Clark et al., 2010; Cheung et al., 2012). Should our hypothesis be supported, our results would suggest that cerebral structures are involved in both the execution and recalibration of corrective actions that result from extended exposure to novel environmental demands.

## Materials and Methods

### Subjects

We tested 16 stroke survivors in the chronic phase (>6 months) with unilateral supratentorial lesions (i.e., without brainstem or cerebellar lesions; age, 62 ± 9.9 years; 6 females; Table 1) and 16 age- and gender-matched control subjects (age, 61 ± 9.7 years; 6 females). We applied the following inclusion criteria: (1) be able to walk with or without a hand-held device at a self-paced speed for at least 5 min; (2) have no orthopedic or pain conditions interfering with the assessment; (3) have no neurologic conditions except stroke; (4) have no severe cognitive impairments (defined as mini-mental state examination score <24); (5) have no contraindications for performing moderate intensity exercise; and (6) use no medication that interferes with cognitive function. We excluded from data analysis 4 of the 32 participants invited for testing. One stroke participant (P7) was excluded because of severe muscle atrophy and weakness on the sound limb (i.e., nonparetic side), which was present prior to the brain lesion. Another stroke participant (P3) was excluded because of poor muscle recordings due to technical difficulties during testing. One control participant (C1) was excluded because this person failed to follow the testing instructions. Last, we had to remove C7 (i.e., age-matched control of P7) because our regression analyses required equal sample sizes across groups. Namely, including fewer participants in the regression of one group reduces the regressor estimates due to more noise in the averaged data. The study protocol was approved by the Institutional Review Board at the University of Pittsburgh. All study participants gave written informed consent prior to participation.

**Table 1:** Clinical characteristics of stroke survivors

Subject	Age (years)	Gender	Affected side	Lesion location	Fugl-Meyer score	Medium walking speed	Adapt strides	Poststrides
**P1**	**43**	**Female**	**R**	**Left MCA and basal ganglia**	**33**	**1.13**	**907**	**605**
**P2**	**64**	**Female**	**R**	**Left MCA and ACA, temporal lobe, basal ganglia**	**26**	**0.81**	**867**	**642**
**P3**	64	Female	R	Left MCA, frontal, parietal lobe and basal ganglia	29	0.60	616	308
**P4**	58	Female	R	Left medial, frontal and parietal area’s	21	0.45	901	624
**P5**	**56**	**Female**	**L**	**Right parietal posterior and temporal lobes**	**31**	**0.94**	**941**	**615**
*P6*	*64*	*Male*	*L*	*Right MCA*	*31*	*0.34*	*452*	*300*
**P7**	78	Male	L	Right MCA			486	217
**P8**	**55**	**Female**	**L**	**Right MCA**	**23**	**0.87**	**903**	**602**
**P9**	**66**	**Male**	**R**	**Left MCA, frontal, temporal and parietal lobes**	**30**	**0.77**	**605**	**599**
**P10**	**60**	**Female**	**R**	**Left frontal**	**26**	**0.9**	**908**	**600**
*P11*	*77*	*Male*	*R*	*Thalamus*	*30*	*0.35*	*590*	*601*
*P12*	*59*	*Male*	*R*	*Left MCA*	*32*	*0.7*	*905*	*600*
***P13***	***52***	***Male***	***R***	***Left MCA***	***32***	***0.96***	***903***	***603***
***P14***	***66***	***Male***	***L***	***Right frontal superior, parietal and posterior area steady state***	***29***	***0.76***	***909***	***602***
***P15***	***75***	***Male***	***R***	***Left periventricular, temporal and basal ganglia***	***32***	***0.94***	***913***	***552***
***P16***	***49***	***Male***	***R***	***Frontotemporal parietal***	***33***	***0.71***	***931***	***303***

ACA, anterior cerebral artery; MCA, middle cerebral artery. Clinical characteristics of stroke survivors. Bold type indicates subjects who are included in the speed-matched analysis. Italic type indicates subjects who are included in the asymmetry-matched analysis. Bold-italic type indicates subjects who are included in both speed-matched analysis and asymmetry-matched analysis.

### Experimental setup and protocol

**Figure 1.**
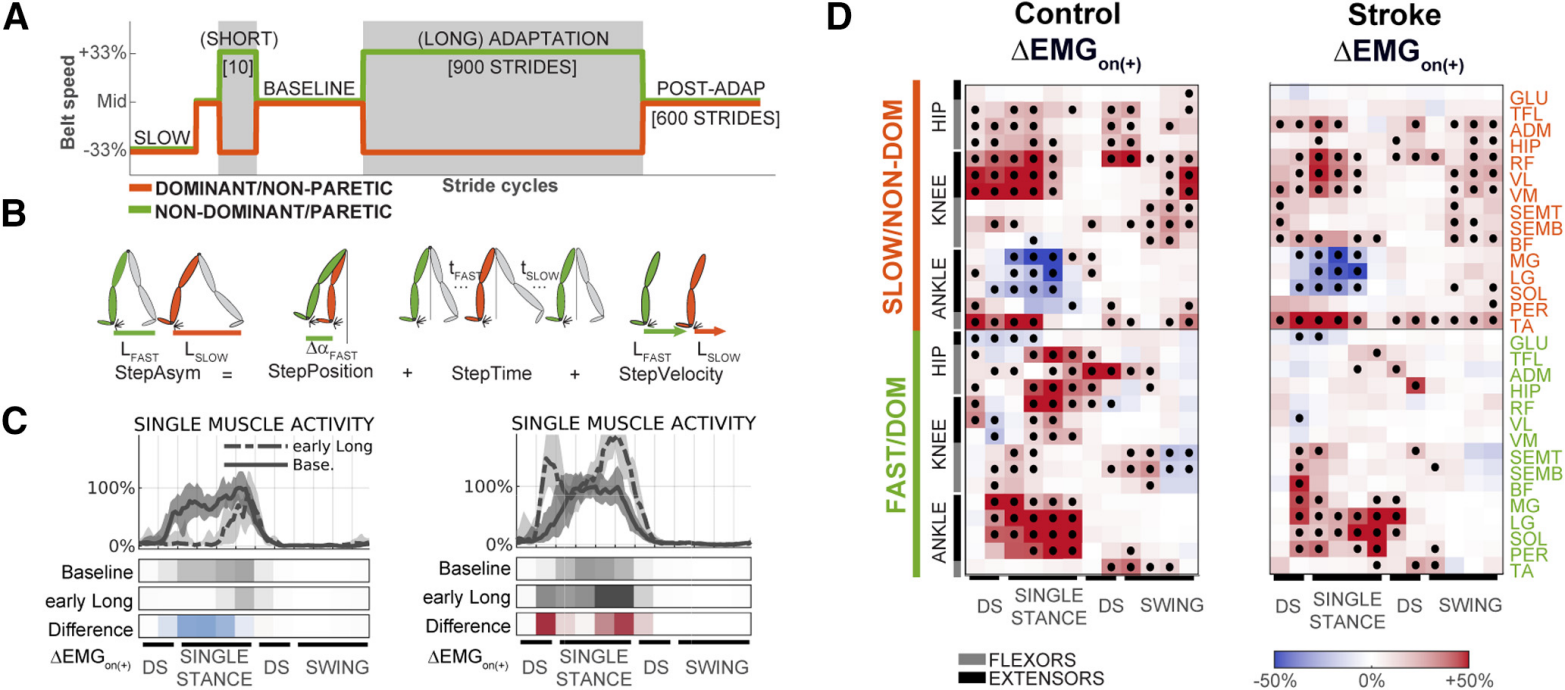
Overview of experimental methods. ***A***, Schedule of belt speeds experienced by subjects. ***B***, Schematic representation of definitions of kinematic parameters StepAsym, StepPosition, StepTime, and StepVelocity, adapted from Sombric et al. (2017). ***C***, Sample EMG traces of one muscle (LG) during baseline and late adaptation for a representative control subject. Median activity across strides (lines), and the 16–84 percentile range (shaded). Data were low-pass filtered for visualization purposes. Color bars below the traces represent averaged normalized values during 12 kinematically aligned phases of the gait cycle (see Materials and Methods) for baseline, early adaptation, and the difference (red indicates increase; blue indicates decrease). ***D***, Corrective responses on introduction of the (+) environment. Left, Control subjects. Right, Stroke survivors. Colors represent group median increase (red) or decrease (blue) in activity. Black dots indicate statistical significance.

We investigated how participants adapted their kinematic and muscle activation patterns on an instrumented split-belt treadmill (Bertec) with two belts that moved at either the same speed (tied condition) or at different speeds (split condition). We first measured subjects’ overground walking speed using the 6 min walking test (Rikli and Jones, 1998; Kervio et al., 2003), and we then performed the Fugl-Myer assessment (Fugl-Meyer et al., 1975). Subsequently, subjects walked on the split-belt treadmill. We kept the mean speed across the belts constant in the tied and split conditions. Each subjects’ mean belt speed was set to 0.35 m/s below their overground walking speed during the 6 min walking test, yielding a comfortable speed for treadmill walking. The mean belt speed, denoted as medium speed, is reported for each subject in Table 1. In the split condition, the speed of one belt was decreased (slow belt) and the speed on the other belt was increased (fast belt) by 33% of the medium speed to obtain a belt speed ratio of 2:1. Stroke survivors walked with their paretic leg on the slow belt, whereas healthy subjects walked with their nondominant leg on the slow belt. The treadmill protocol consisted of five periods: (1) 50 strides (i.e., time between two subsequent heel-strikes of the same leg) walking at medium speed to familiarize subjects with treadmill walking; (2) a short exposure (10 strides) to the split condition to allow subjects to briefly experience the split condition prior motor adaptation; (3) 150 strides of baseline walking at medium speed to characterize subjects’ baseline gait; (4) 900 strides of adaptation to the split condition, which is a long enough period for locomotor adaptation (Iturralde and Torres-Oviedo, 2019); and (5) 600 strides for afteradaptation at medium speed to measure adaptation effects (i.e., aftereffects) and their decay (Fig. 1*A*). Subjects had several resting breaks during the experiment and some stroke individuals completed fewer strides during adaptation and afteradaptation to prevent fatigue (Table 1 shows the number of strides completed per subject). Participants wore a safety harness, not supporting body weight, attached to a sliding rail in the ceiling to prevent falls. Moreover, subjects could hold on to a handrail in front of the treadmill, but were instructed to do so only if needed.

### Data collection

We collected kinetic, kinematic, and electromyogram (EMG) data to characterize individuals’ walking pattern. The ground reaction force aligned with gravity (Fz; sampled at 1000 Hz) was used to identify the instants at which the feet landed (i.e., heel-strike: Fz > 10 N) or were lifted from the ground (i.e., toe-off: Fz < 10 N; Iturralde and Torres-Oviedo, 2019). The positions of the ankles (lateral malleolus) and hips (greater trochanter) were recorded at 100 Hz using a 3D motion analysis system (Vicon Motion Systems). The activity of 15 muscles was recorded bilaterally at 2000 Hz using a Delsys Trigno System (Delsys): gluteus medius, tensor fasciae latae, adductor magnus, hip flexors, rectus femoris, vastus lateralis, vastus medialis, semitendinosus, semimembranosus, biceps femoris, gastrocnemius medialis, gastrocnemius lateralis (LG), soleus, peroneus, and tibialis anterior. EMG signals were high-pass filtered with a 30 Hz fourth-order Butterworth dual-pass filter and subsequently rectified (Merletti and Parker, 2005).

### Data analysis

#### Kinematic parameters

We characterized the adaptation of step-length asymmetry (StepAsym; Eq. 1; Fig. 1*B*), which is conventionally used to quantify gait changes during split-belt walking (Reisman et al., 2007; Torres-Oviedo et al., 2011). We defined StepAsym as the difference between consecutive steps of the legs in terms of step length, where step length is the distance between the feet (i.e., ankle markers) at heel-strike. In our definition, StepAsym is positive when the step length of the fast leg (i.e., dominant or nonparetic) is larger than the one of the slow leg (nondominant or paretic). We also quantified spatial (StepPosition) and temporal (StepTime) gait features that contribute to StepAsym, since those are differentially affected across stroke survivors, and they exhibit distinct adaptation patterns in unimpaired adults during split-belt walking (Finley et al., 2015). Finally, StepVelocity was defined as the difference between the legs in terms of velocity of the foot with respect to the body when in contact with the ground. All parameters were expressed in units of distance, and they were normalized to the sum of left and right step lengths to account for differences in step sizes across subjects (Sombric et al., 2017).

#### EMG parameters

We characterized the modulation of muscle activity across the different walking conditions using the average activity of each muscle for fixed phases of the gait cycle (Fig. 1*C*). Specifically, we divided the gait cycle into the following four phases: first double support (DS; between ipsilateral heel-strike and contralateral toe-off), single stance (SINGLE; from contralateral toe-off to contralateral heel-strike); second DS (between contralateral heel-strike and ipsilateral toe-off) and swing (SWING; between ipsilateral toe-off and ipsilateral heel-strike). We further divided each of these phases to achieve better temporal resolution. Specifically, both DS phases were divided in two equal subphases, and the SINGLE and SWING phases were subdivided in four equal subphases. Muscle activity amplitude was averaged in time for each of these subintervals for every stride and muscle, resulting in 180 muscle activity variables per leg per stride cycle: 12 subinterval × 15 muscles.

EMG activity for each muscle was linearly scaled to baseline walking (last 40 strides), such that a value of 0 corresponded to the average of the interval with the lowest average activity and 1 corresponded to the average of the interval with the highest average activity (Iturralde and Torres-Oviedo, 2019). This normalization enabled us to aggregate the EMG activity across subjects to perform group analyses. Of note, we excluded from analysis the activity of soleus from one stroke survivor because technical difficulties during data collection.

#### Epochs of interest

Kinematic and EMG parameters were used to characterize subjects’ behavior at the beginning (“early”) and at the end (“late”) of each experimental condition. Specifically, the epochs of interest included the following: late baseline walking, early and late adaptation, and early afteradaptation. The early epochs were characterized by the median of the initial five strides and late epochs by the median of the last 40 strides of the condition of interest. We chose medians across strides, rather than means to minimize the impact of outlier values. In all cases, we excluded the very first and very last stride of each condition to avoid artifacts from starting and stopping the treadmill. Subsequently, we subtracted the late baseline behavior from all epochs of interest. This allowed us to identify group differences in subjects’ modulation of kinematic and EMG parameters beyond those due to distinct baseline biases. Moreover, we computed the differences between EMG activity early afteradaptation versus late adaptation to quantify changes in EMG activity on sudden removal of the perturbation.

#### Sensorimotor recalibration of corrective muscle responses

**Figure 2.**
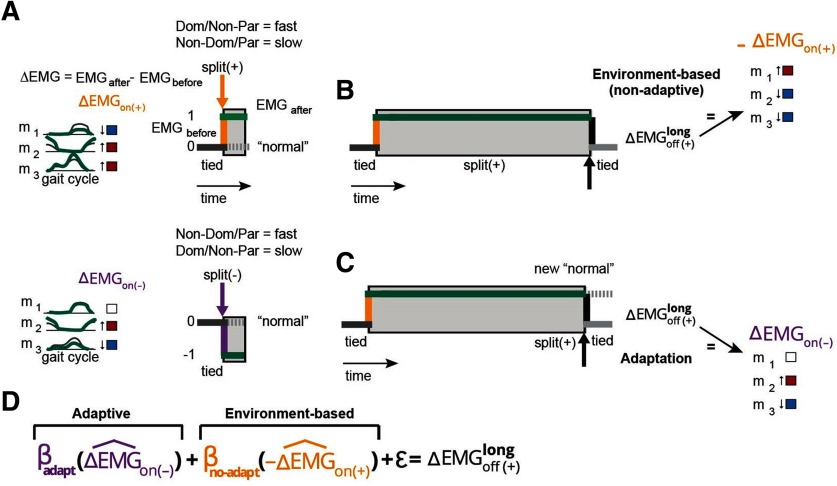
Environment-based and adaptive contributions to corrective responses. ***A***, Schematic representation of corrective responses on the introduction split-belt perturbation. The split environment is arbitrarily defined as + if the paretic leg is on the slow belt and the nonparetic leg is on the fast belt (top cartoon), whereas it is defined as − if the paretic leg is on the fast belt and the nonparetic leg is on the slow belt. Changes in muscle activity (i.e., corrective response) on the introduction of the + or − environment are color coded as blue (decreased activity), red (increased activity), and white (no change in activity). ***B***, In the case of an environment-based corrective response changes in muscle activity perturbation removal ($\Delta {\text{EMG}}_{\text{off}(+)}$) are opposite to those on perturbation introduction. ***C***, In the case of an adaptive corrective response, the split environment is perceived as the new normal. Consequently, removal of the split environment will be experienced as a perturbation in the opposite direction. Thus, the structure of the corrective response will resemble the one observed on introduction of the − environment. ***D***, Regression equation used to quantify the structure of corrective response $\Delta {\text{EMG}}_{\text{off}(+)}$. In this equation, β_adapt_ quantifies the similarity of $\Delta {\text{EMG}}_{\text{off}(+)}$ to the adaptation-based response and β_no-adapt_ quantifies the similarity of $\Delta {\text{EMG}}_{\text{off}(+)}$ to the environment-based response.

We studied the structure (i.e., activity across multiple muscles) of corrective motor responses on sudden changes in the walking environment (Fig. 2), since this reflects the extent of sensorimotor recalibration (Iturralde and Torres-Oviedo, 2019). We defined corrective responses as the rapid changes in motor output (ΔEMG) immediately after a transition in the walking environment. Corrective responses were quantified as the difference in muscle activity immediately after an environmental transition (EMG_after_) compared with the muscle activity before the transition (EMG_before_; Fig. 2*A*). Thus, corrective response (ΔEMG) = EMG_after_ – EMG_before_. Since we had multiple strides before and after a transition, we used the median EMG activity across either 40 or 5 strides to quantify EMG_before_ and EMG_after_ a given transition, respectively.

Corrective responses, ΔEMG, were labeled according to the environmental transition and split-belt environment that was inducing them. More specifically, ΔEMG_on_ referred to corrective responses when the split environment was introduced (“on” transition), and ΔEMG_off_ referred to those when the split environment was removed (“off” transition). Also, corrective responses were labeled “(+)” or “(−)” to indicate the specific split environment that was generating them. Specifically, in the (+) environment, the dominant leg, or the nonparetic leg in patients, walked faster than the other leg, whereas in the (−) environment the nondominant leg, or the paretic leg in patients, walked faster than the other leg (Fig. 2*A*). Thus, $\Delta {\text{EMG}}_{\text{on}(+)}$ was computed as the difference between EMG activity before and after the (+) environment was introduced.

We were specifically interested in the structure of corrective responses postadaptation because this structure indicates the extent to which subjects recalibrate their motor system (Iturralde and Torres-Oviedo, 2019). Namely, the structure of these corrective responses is determined by both changes in the environment and changes in the motor systems’ adaptive state. We discerned the environment-based and adaptive-based contributions to corrective responses afteradaptation ($\Delta {\text{EMG}}_{\text{off}(+)}$) with a regression model ($\Delta {\text{EMG}}_{\text{off}(+)}$ = adaptive-based + environment-based + ε). In the case of an environment-based response, the corrective pattern $\Delta {\text{EMG}}_{\text{on}(+)}$ on introducing the ‘+ split environment is simply disengaged once this environment is removed (i.e., both belts moving at the same speed; Iturralde and Torres-Oviedo, 2019). Thus, in this case the structure of corrective responses afteradaptation $\Delta {\text{EMG}}_{\text{off}(+)}$ (i.e., when the split ‘+’ environment is turned off) resembles the numerical opposite of $\Delta {\text{EMG}}_{\text{on}(+)}$ ($\Delta {\text{EMG}}_{\text{off}(+)}$ = −$\Delta {\text{EMG}}_{\text{on}(+)}$; Fig. 2*B*). Conversely, adaptive-based responses are observed if subjects perceive the split-belt environment (+) as the “new normal.” Consequently, removing the (+) environment is processed as a perturbation in the opposite direction as the one originally experienced [i.e., it would be equivalent to introduction of the (−) environment; Fig. 2*A*]. Thus, in the case of adaptive corrective responses, the structure of $\Delta {\text{EMG}}_{\text{off}(+)}$ resembles corrective responses to transitioning into the opposite (−) split-belt environment ($\Delta {\text{EMG}}_{\text{off}(+)}$ = $\Delta {\text{EMG}}_{\text{on}(-)}$; Fig. 2*C*). Note that the corrective responses afteradaptation ($\Delta {\text{EMG}}_{\text{off}(+)}$) exhibit features of both environment-based and adaptive-based responses. Thus, we used a regression analysis to determine the extent to which the structure of corrective responses afteradaptation was environment-based or adaptive-based (Fig. 2*D*), as follows:

$${\hat{\Delta \text{EMG}}}_{\text{off(}+\text{)}}{\text{=-}\beta }_{\text{no-adapt}}{\hat{\Delta \text{EMG}}}_{\text{on(}+\text{)}}{+\beta }_{\text{adapt}}{\hat{\Delta \text{EMG}}}_{\text{on(}-\text{)}}+\text{ε}$$

In the regression equation, the parameters β_no-adapt_ and β_adapt_ are respectively interpreted as the extent to which the structure of corrective responses indicates transitions in the environment (i.e., environment based) or the adaptation of subjects’ motor system (i.e., adaptive based). Note that every vector is divided by its norm (i.e., ${\hat{\Delta \text{EMG}}}_{\text{off(}+\text{)}}\;\text{=}\;\Delta {\text{EMG}}_{\text{off}\left(+\right)}\text{/}\left\|\Delta {\text{EMG}}_{\text{off}\left(+\right)}\right\|$). This was done because we were interested in identifying stroke-related deficits in the structure, rather than the magnitude of corrective responses, which is known to be different (De Kam et al., 2017). For example, we find that the amplitude of corrective responses, $\Delta {\text{EMG}}_{\text{on}(+)}$, for each leg was smaller for the stroke group (${\hat{\Delta \text{EMG}}}_{\text{on(}+\text{)}}=$ 2.6 and 2.2) than the control group (3.3 and 3.7).

Note that $\Delta {\text{EMG}}_{\text{on}(-)}$ was not directly measured to avoid exposing subjects to multiple environmental transitions prior to the adaptation period. Instead, we inferred these responses by exploiting the symmetry of the transition between the two legs. The only difference between the + and − environments is which leg increases speed and which leg decreases it. We used this similarity to infer the (not recorded) corrective responses ($\Delta {\text{EMG}}_{\text{on}(-)}$) of each leg to transitioning into the − environment from the (measured) corrective responses ($\Delta {\text{EMG}}_{\text{on}(+)}$) to transitioning to the + environment. In other words, we assumed that the (not recorded) nondominant leg’s responses to the “on (−)” transition would be similar to the (recorded) dominant leg’s responses to the “on (+)” transition, and vice versa. We are aware that this assumption might not be valid for some afterstroke individuals, given their inherent motor asymmetry. Thus, group differences in β_adapt_ values, which are estimated using the not recorded $\Delta {\text{EMG}}_{\text{on}(-)}$ in our regression analysis, might be due to the experimental limitation of our study. To address this possibility, we performed a *post hoc* analysis to compare the regression coefficients between a subset of patients and control subjects (*n* = 7 on each subgroup) that had similar asymmetry in their EMG activity during baseline walking (*p* = 0.1). The baseline asymmetry in EMG activity across the legs was quantified in each subject by first computing a 180-dimension vector (15 muscles × 12 gait cycle phases) of the baseline muscle activity for each leg and then calculating the cosine between those baseline vectors for the legs of each individual.

#### Structure of muscle activity patterns in a novel walking environment

We characterized changes in the structure of steady-state muscle activity from baseline walking to late adaptation (ΔEMG_SS_= EMG_late adaptation − EMGlate baseline_). This was defined as the pattern of activity across all muscles and all gait cycle intervals (15 muscles × 12 intervals = 180 data points for a given epoch). The $\Delta {\text{EMG}}_{\text{SS}}$ 180-dimensional vector for each subject was used to assess structural differences between stroke survivors and control subjects. We specifically computed a cosine between the $\Delta {\text{EMG}}_{\text{SS}}$ for each individual and a “reference pattern” $\Delta {\text{EMG}}_{\text{SS}}$, which was defined as the median $\Delta {\text{EMG}}_{\text{SS}}$ of the control group. This reference pattern for $\Delta {\text{EMG}}_{\text{SS}}$ was calculated as the group median of all control subjects when computing the similarity metric for each leg of the stroke survivors, whereas for individual control subjects we excluded the subjects’ own data to compute the reference vector. A cosine closer to 1 indicates that the subject-specific and “reference” vectors are more aligned, and therefore, the structures of the muscle patterns that they represent are similar.

### Statistical analyses

#### Modulation of muscle activity within groups

Modulation of muscle activity was first evaluated for each group individually. Specifically, we compared muscle activity between the epochs of interest using a Wilcoxon signed-rank test (nonparametric equivalent of paired *t* test) for each individual muscle and for each gait cycle phase, resulting in 360 comparisons per epoch (12 intervals × 15 muscles × 2 legs). We subsequently corrected the significance threshold for each epoch using a Benjamini–Hochberg procedure (Benjamini and Hochberg, 1995) to indicate significant changes in our figures, but all data in both groups was used in the structural analyses.

#### Structure of muscle activity patterns during steady-state walking

We used a Wilcoxon rank sum test to compare the groups on their $\Delta {\text{EMG}}_{\text{SS}}$ for each leg during late adaptation in the split-belt condition. We specifically compared the group’s similarity in $\Delta {\text{EMG}}_{\text{SS}}$ to the reference pattern obtained with the cosine analysis.

#### Sensorimotor recalibration of corrective muscle responses

We compared the regressor coefficients β_no-adapt_ and β_adapt_ for each group to determine whether stroke survivors and control subjects differed in the adaptation of corrective responses. Since the regressor estimates of β_no-adapt_ and β_adapt_ in a regression model are not independent, between-group comparisons were performed in the 2D space covered by β_no-adapt_ and β_adapt_. The differences between the groups were compared using a χ^2^ distribution, which could be considered as a high-dimensional *t* test (Härdle and Simar, 2007).

#### Correlation analyses

We asked whether individual subjects’ adaptation of muscle activity was related to the severity of motor impairment (i.e., Fugl-Meyer score). To this end, we performed Spearman correlations between (1) the Fugl-Meyer score and (2) outcome measures that reflected sensorimotor recalibration (i.e., β_adapt_ and β_no-adapt_) and the similarity metric comparing the structure of muscle activity during late adaptation in the split-belt condition for each individual versus a reference $\Delta {\text{EMG}}_{\text{SS}}$.

#### Modulation of kinematic parameters

We compared stroke survivors and control subjects in how they modulated kinematic parameters. To this end, we performed a repeated-measures analysis of variance for each kinematic outcome (StepAsym, StepPosition, StepTime, and StepVelocity) with GROUP (stroke vs control subjects), EPOCH (early adaptation, late adaptation, and early postadaptation), and the interaction between both variables as predictors. Note that we did this analysis with unbiased data (i.e., baseline subtracted) because we were interested in differences in modulation across groups, beyond their baseline biases. In case of a significant GROUP or GROUP × EPOCH interaction effect, we performed between-group comparisons for each epoch using Bonferroni-corrected independent *t* tests (adjusted α = 0.017).

#### Speed-matched *post hoc* analysis

We found that stroke survivors walked slower than control subjects during the experiment (averaged medium speed = 0.78 ± 0.24 vs 1.07 ± 0.12 m/s; rank-sum test, *p* < 0.01), which could confound between-group differences in muscle activity. Thus, we repeated our analyses with only the 10 fastest participants in the stroke group and the 10 slowest control subjects to determine whether structural differences between our groups were due to walking speed, rather than brain lesion. Walking speed was not significantly different for these speed-matched subgroups (0.88 ± 0.18 vs.1.0 ± 0.15 m/s; rank sum test, *p* = 0.10). Importantly, the selection of the fastest stroke survivors did not result in a selection of patients with less severe motor impairments (Fugl-Meyer score = 29.5 ± 3.4 vs 28.5 ± 5.1; *p* = 0.67, for subgroup included vs subgroup excluded in the speed-matched comparison, respectively).

## Results

### Cerebral lesions interfered with the structure of muscle activity in a novel walking environment

**Figure 3.**
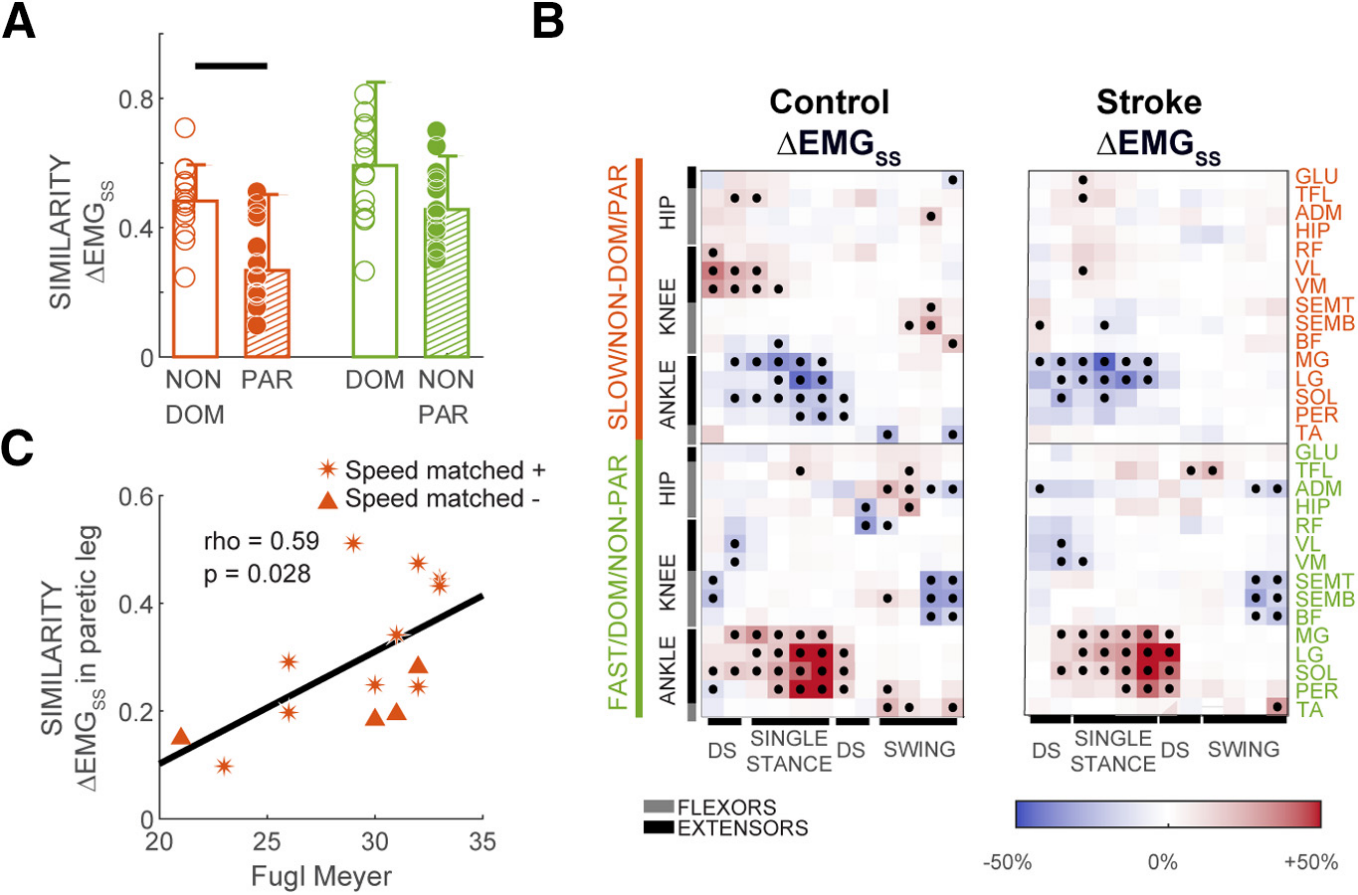
Structure of muscle activity modulation. ***A***, Similarity of individual subjects’ steady-state muscle activity modulation to the reference pattern (i.e., expressed as the cosine between individual subject vector and group median of control subjects). Values closer to 1 indicate more similarity between vectors. Bars indicate group medians, and error bars represent the interquartile range. Horizontal lines indicate significant differences in group medians as determined with a Wilcoxon rank sum test (*p* < 0.05). ***B***, Visual representation of the structure of muscle activity modulation in the steady state of split-belt walking ($\Delta {\text{EMG}}_{\text{SS}}$) relative to baseline walking. Red colors indicate increased activity, and blue colors indicate decreased activity. Dots indicate statistical significance for nonparametric within-group comparisons. We corrected the significance threshold for each epoch using a Benjamini–Hochberg procedure (Benjamini and Hochberg, 1995), setting the acceptable false discovery rate to 10%. In addition, we focused on significant differences between epochs that exceeded 10% of the maximum baseline activity for a given muscle since we considered these to be meaningful changes. Corrected *p* thresholds for $\Delta {\text{EMG}}_{\text{SS}}$ were 0.058 for control subjects and 0.02 for stroke. ***C***, Association between severity of motor symptoms (Fugl-Myer test) and structure of EMG modulation in steady-state walking ($\Delta {\text{EMG}}_{\text{SS}}$). Asterisks represent subjects included in the speed-matched analysis, whereas triangles indicated subjects that were excluded. We found a significant correlation (i.e., Spearman’s ρ). This correlation indicated that stroke survivors who were less severely affected (i.e., higher Fugl-Myer score) exhibited a steady-state muscle pattern that was more similar to that of the reference muscle pattern (which was computed using EMG recordings from intact subjects).

We computed a similarity metric, $\Delta {\text{EMG}}_{\text{SS}}$, which indicated the similarity between the structure of an individual’s muscle activity modulation in steady-state walking relative to the average pattern in control subjects (reference pattern). We found that the nonparetic leg activity at steady state was similar to the one of control subjects, whereas the paretic leg was not (Fig. 3*A*). Differences in the structure of muscle activity modulation between the groups can be appreciated in Figure 3*B*. Specifically, similarity metric $\Delta {\text{EMG}}_{\text{SS}}$ was lower in the paretic leg compared with control subjects (Fig. 3*A*; *p* = 0.001), and between-group differences were trending (*p* = 0.057) when comparing the nonparetic leg activity to that of control subjects. These between-group differences were not observed when patients and control subjects walked at similar speeds (median ± interquartile range in control subjects vs stroke survivors for the nonparetic leg: 0.58 ± 0.26 vs 0.51 ± 0.22, *p* = 0.47; paretic leg: 0.39 + 0.13 vs 0.28 ± 0.18, *p* = 0.1). Interestingly, a more atypical structure in muscle activity modulation in the paretic leg was associated with poorer voluntary leg motor control as measured by the Fugl-Meyer scale (ρ = 0.59, *p* = 0.028, Fig. 3*C*), but not in the nonparetic leg (ρ = −0.29, *p* = 0.32; data not shown). In conclusion, the structure of muscle activity at steady state was different between patients and control subjects, and individuals with more atypical paretic activity were those with lower voluntary function.

### Sensorimotor recalibration of corrective responses was intact after cerebral lesion

**Figure 4.**
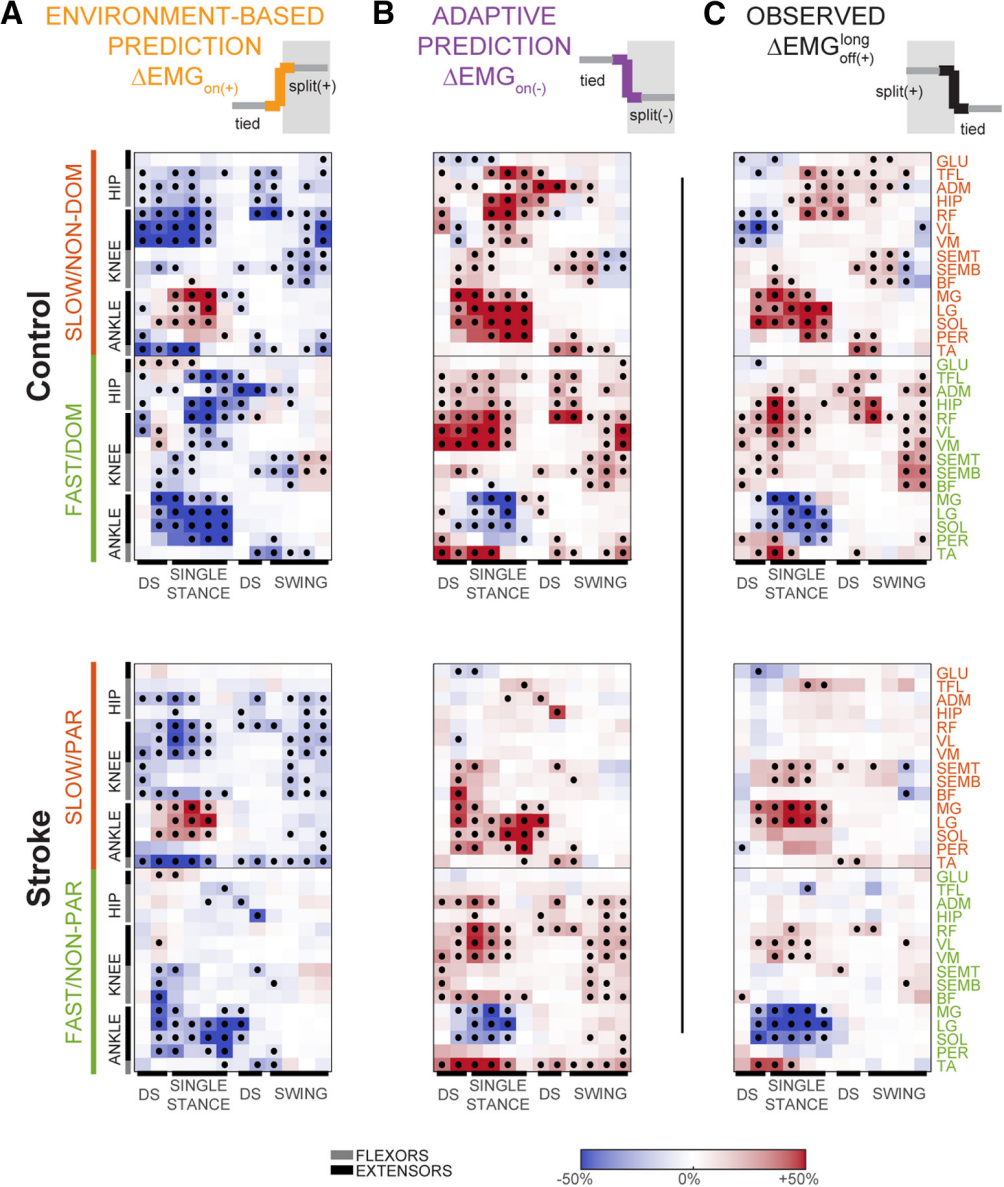
Predicted and measured structure of corrective responses after a long adaptation period. Data of control subjects are shown in the top panels and those of the stroke participants in the bottom panels. ***A***, ***B***, Expected corrective responses elicited by the off transition under the environment-based (***A***) and adaptive (***B***) cases. Data (in color) and significance (black dots) were derived from the observed corrective responses on the introduction of the (+) walking environment, by either taking the numerical opposite (environment-based) or by transposing leg activity (adaptation-based). ***C***, Measured corrective responses on removal of the (+) environment.

**Figure 5.**
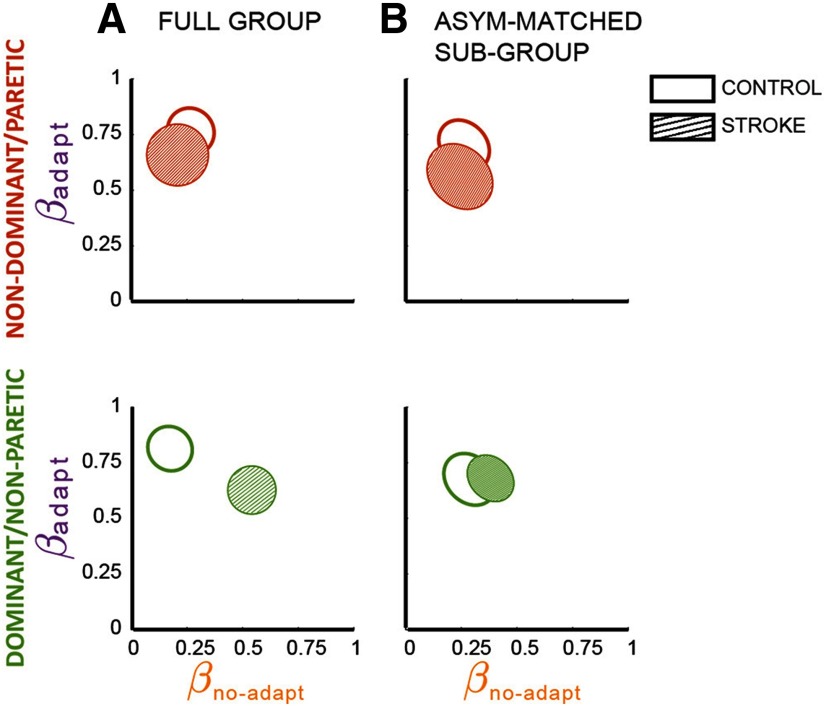
Adaptive and environment-based contributions to corrective responses. The ellipses represent the regression estimations of β_adapt_ and β_no-adapt_ and their 95% confidence intervals for the control group (open) and the stroke group (hatched). ***A***, Data were obtained with 14 subjects/group. Paretic leg: *R*^2^ = 0.47, model *p* = 8.8 * 10^−26^; nonparetic leg: *R*^2^ = 0.67, model *p* = 1.3 * 10^−45^; dominant leg in control subjects: *R*^2^ = 0.71, model *p* = 1.1 * 10^−48^; nondominant leg in control subjects: *R*^2^ = 0.68, model *p* = 3.1 * 10^−45^. ***B***, Data obtained for asymmetry-matched groups (i.e., *n* = 7/group). Nondominant/paretic leg: control subjects: CI for β_adapt_ = 0.61–0.79; CI for β_no-adapt_ = 0.17–0.25; *R*^2^ = 0.64; model *p* = 1.2 * 10^−40^; stroke: CI for β_adapt_ = 0.45–0.68; and CI for β_no-adapt_ = 0.13–0.36; *R*^2^ = 0.44; model *p*  = 6.2*10^−23^; between-group comparison: χ^2^ = 4.1, *p* = 0.13; dominant/nonparetic leg: control subjects: CI for β_adapt_ = 0.58 0.77; CI for β_no-dapt_ = 0.20–0.38; *R*^2^ = 0.63; model *p* = 8.4 * 10^−39^; stroke: CI for β_adapt_ = 0.60–0.76; CI for β_no-adapt_ = 0.30–0.46; *R*^2^ = 0.73; model *p* = 8.8 * 10^−51^; between-group comparison: χ^2^ = 2.5, *p* = 0.29.

The structure of corrective responses for each group indicated that on average both groups recalibrated their gait similarly. This is qualitatively indicated by the “checker boards” illustrated in Figure 4. Notice that in both groups the observed corrective responses postadaptation (Fig. 4*C*) look more similar to those predicted by the adaptive (Fig. 4*B*) than the environment-based modulation (Fig. 4*A*). The environment-based and adaptive-based contributions to corrective responses postadaptation were quantified with a regression model, which reproduced the data well (Fig. 5, left panels). We observed that the regression coefficient β_adapt_ was greater than β_no-adapt_ in both groups for the leg that walked slow (i.e., nondominant leg in control subjects: CI for β_adapt_ = 0.68–0.85 vs CI for β_no-adapt_ = 0.18–0.35; paretic leg in stroke: CI for β_adapt_ = 0.55–0.77 vs CI for β_no-adapt_ = 0.10–0.32) and the leg that walked fast (i.e., dominant leg in control subjects: CI for β_adapt_ = 0.73–0.89 vs CI for β_no-adapt_ = 0.09–0.25; nonparetic leg in stroke: CI for β_adapt_ = 0.54–0.71 vs CI for β_no-adapt_ = 0.46–0.62). These coefficients were not different between groups when estimated from the averaged paretic leg activity across stroke survivors vs that of the nondominant leg across control subjects (χ^2^ = 3.2, *p* = 0.20), indicating that averaged responses in the slow leg were adapted to the same extent in stroke survivors and control subjects. Conversely, we found between-group differences when comparing the coefficients of the averaged nonparetic activity in the stroke group versus that of the dominant leg in the control group (χ^2^ = 48.9, *p* = 2.4 * 10^−11^; Fig. 5*A*, bottom). Thus, we observed between-group differences in the regression coefficients for the nonparetic, but not the paretic, compared with control legs.

As a *post hoc* analysis, we considered the possibility that these group differences in the nonparetic side could arise from our estimation of the adaptive-based modulation ($\Delta {\text{EMG}}_{\text{on}(-)}$). Notably, this muscle activity was not recorded but it was inferred from the muscle activity of the other leg, assuming symmetry of corrective responses across legs. Given that stroke survivors exhibit asymmetric motor patterns, the $\Delta {\text{EMG}}_{\text{on}(+)}$ of the paretic leg may not be a good estimate for the $\Delta {\text{EMG}}_{\text{on}(-)}$ of the nonparetic leg, thereby leading to underestimation of β_adapt_ in this leg. Thus, we performed a subgroup analysis in which stroke survivors and control subjects were matched for symmetry in their muscle activity during baseline walking. We did not find between-group differences for either leg of the stroke group compared with the control subjects when asymmetry in baseline muscle activity was matched between the groups (Fig. 5*B*; paretic vs nondominant control leg: χ^2^ = 4.1, *p* = 0.13; nonparetic vs dominant control legs, χ^2^ = 2.5, *p* = 0.29). In conclusion, the observed structure of corrective responses postadaptation were more similar to the one predicted by adaptive, rather than environment-based, modulation in patients with cerebral lesions and control subjects.

**Figure 6.**
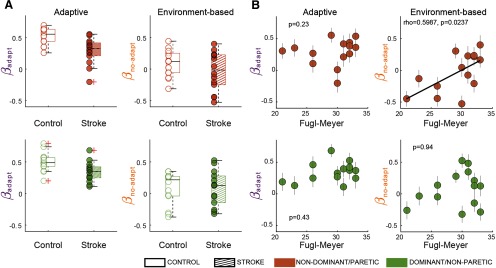
Individual regression results. ***A***, Intersubject variability for the adaptive (β_adapt_) and environment-based (β_no-adapt_) contributions to corrective responses in the slow/paretic leg (top panels) and the fast/nonparetic leg (bottom panels). Median ± interquartile range for regressors are as follows: nondominant leg: β_adapt_ = 0.55 ± 0.19; β_non-adapt_ = 0.12 ± 0.30; *p* = 9.2 * 10^−19^ ± 7.7 * 10^−13^; dominant leg: β_adapt_ = 0.49 ± 0.14; β_non-adapt_ = 0.22 ± 0.31; *p* = 1.2 * 10^−15^ ± 4.7 * 10^−12^; paretic leg: β_adapt_ = 0.33 ± 0.21; β_non-adapt_ = −0.02 ± 0.4; *p* = 2.1 * 10^−8^ ± 3.2 * 10^−5^; nonparetic leg: β_adapt_ = 0.35 ± 0.17; β_non-adapt_ = 0.13 ± 0.42; *p* = 1.6 * 10^−9^ ± 6.2 * 10^−6^. ***B***, Spearman correlations between leg motor function (Fugl-Myer scale) and β_adapt_ and β_no-adapt_ for each leg.

While recalibration of corrective responses poststroke did not differ from that of control subjects at the group level when asymmetries were accounted for, we considered the possibility that some individuals would exhibit less recalibration compared with others. Consistently, Figure 6*A* shows a wide range of β_adapt_ and β_non-adapt_ regression values at the individual level. Also, note that the regression model had smaller *R*^2^ values when applied to each subject’s corrective responses postadaptation (control subjects’ nondominant leg: *R*^2^ = 0.38 ± 0.18; control subjects’ dominant leg: 34 ± 0.17; paretic leg: 0.18 ± 18; nonparetic leg: 0.18 ± 18) than to the corrective response of the group (reported in previous section). However, the regression model was significant in all individuals, except for one stroke survivor (*p* = 0.19). In sum, we find large ranges of regression coefficients in control and poststroke individuals.

We further asked whether stroke survivors would exhibit less recalibration if they had more severe leg motor impairments (i.e., Fugl-Meyer scale). Thus, we computed the Spearman correlation between individual subjects’ regressors and their leg motor score (Fig. 6*B*). We found that β_adapt_ of neither the paretic or nonparetic legs was correlated to the Fugl-Meyer score (paretic: ρ = 0.34, *p* = 0.23; nonparetic: ρ = 0.23, *p* = 0.43). On the other hand, motor function measured with the Fugl-Meyer score was associated with β_no-adapt_ for the paretic leg and not the β_no-adapt_ for the nonparetic leg (paretic leg: ρ = 0.60, *p* = 0.024; nonparetic leg: ρ = 0.02, *p* = 0.94). However, this correlation was driven by the individual with the largest negative β_no-adapt_ value since the correlation was no longer significant when this subject was excluded (ρ = 0.37, *p* = 0.22). As such, we are cautious about interpreting this result as a positive association between environment-based corrective response and leg motor scores. Together, our correlation analyses indicate that recalibration of corrective responses is not associated with the quality of voluntary motor control.

### Stroke-related deficits in muscle coordination are not reflected in asymmetry parameters

**Figure 7.**
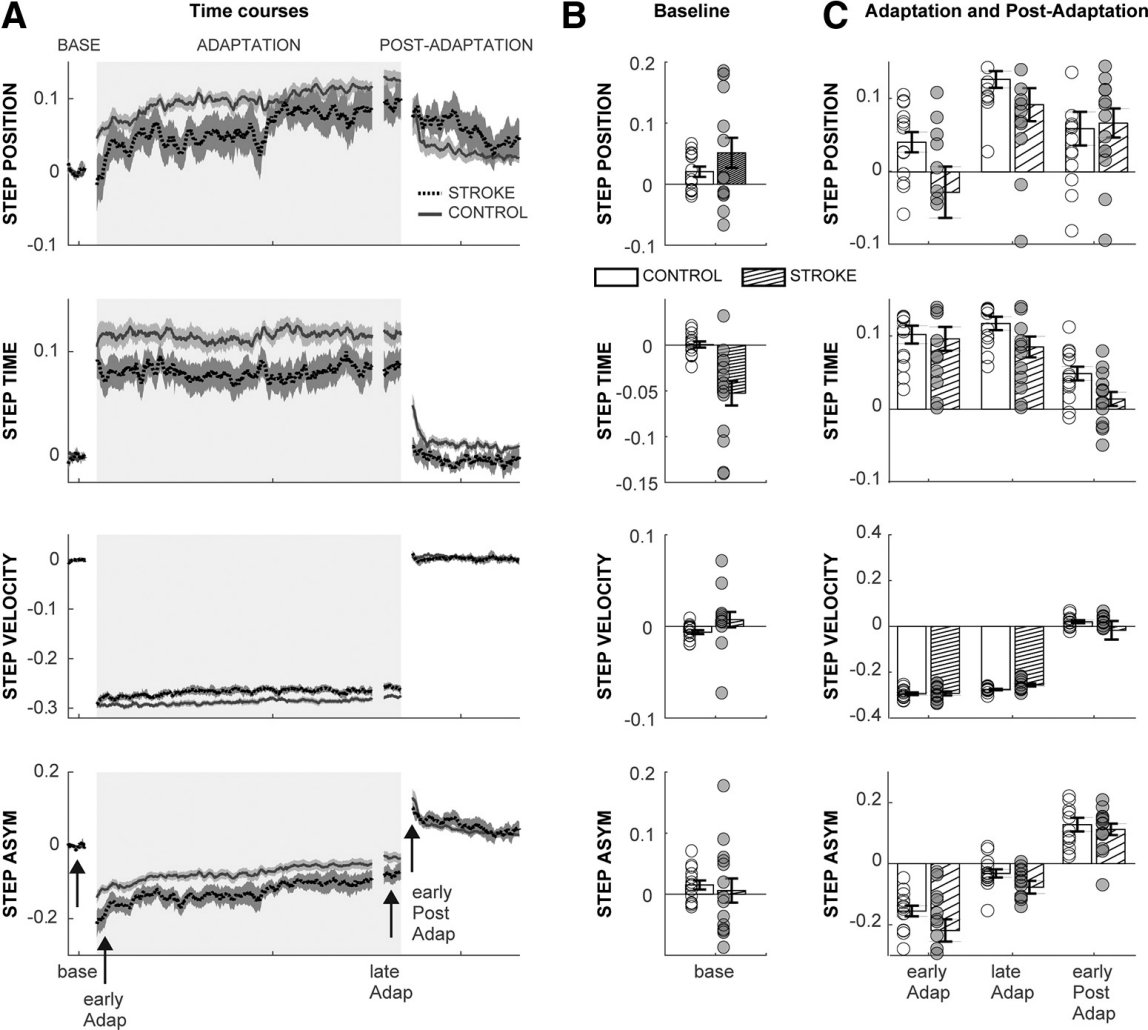
Modulation of kinematic parameters. ***A***, Group-averaged time courses for StepPosition, StepTime, StepVelocity, and StepAsym. Note that individual subjects’ baseline biases were subtracted to allow for comparison of modulation of parameters regardless of differences in baseline asymmetry. Shaded areas represent SEs for each group. For visual purposes, data were smoothed using a running average (median) of 10 strides. Rectangles represent the epochs of interest. ***B***, Interlimb kinematic parameters for each group during baseline. ***C***, Between-group comparisons for kinematic parameters over the epochs of interest. We found no significant differences between the groups in any of the parameters. StepAsym (GROUP: *F*_(1,28)_ = 3.48, *p* = 0.07; GROUP × EPOCH: *F*_(2,56)_ = 1.16, *p* = 0.31), stepPosition (GROUP: *F*_(1,28)_ = 2.14, *p* = 0.16; GROUP × EPOCH: *F*_(2,56)_ = 2.33, *p* = 0.13), stepTime (GROUP: *F*_(1,28)_ = 3.17, *p* = 0.09; GROUP × EPOCH: *F*_(2,56)_ = 0.63, *p* = 0.50), and stepVelocity (GROUP: *F*_(1,28)_ = 1.41, *p* = 0.25; GROUP × EPOCH: *F*_(2,56)_ = 1.11, *p* = 0.34).

While stroke survivors exhibited deficits in the execution of updated motor commands during steady-state split-belt walking (i.e., $\Delta {\text{EMG}}_{\text{SS}}$), we observed no differences between the groups in the modulation of asymmetry parameters (i.e., stepAsym, stepPosition, stepTime, and stepVelocity; Fig. 7). Specifically, we observed no main effects of GROUP or GROUP × EPOCH interaction effects for the interlimb kinematic parameters (*p* > 0.05). Comparable results were obtained in our speed-matched analysis. Thus, interlimb kinematic parameters are less sensitive to stroke-related deficits in locomotor adaptation than our outcome measures for muscle coordination.

## Discussion

We studied the involvement of cerebral structures in the sensorimotor recalibration of gait using stroke as a clinical model. We found that on average stroke survivors exhibit similar recalibration of corrective responses in the paretic leg relative to control subjects, which was surprising given the known deficits in paretic responses poststroke. On the other hand, we found that cerebral lesions affected the muscle activity of paretic legs in the steady state of split-belt walking. Thus, we find an interesting dissociation between execution and recalibration of corrective actions: the execution of motor patterns on novel demands is cerebral dependent, but the recalibration is not. These results do not support our original hypothesis that execution and recalibration of corrective responses would be a cerebral-mediated process. Our findings suggest as though this latter process might only depend on other structures such as the cerebellum.

### Sensorimotor recalibration of corrective responses after cerebral lesions

Our results suggest that while corrective responses are affected by lesions to cerebral structures (Marigold and Eng, 2006; De Kam et al., 2017, 2018) and corticospinal tract (Christensen et al., 1999, 2001), the recalibration of this corrective activity is not strongly mediated by these neural structures. More specifically, we found that the changes in corrective responses postadaptation are similar between individuals with cerebral lesions and control subjects. This is clearly evident in the paretic leg, but not in the nonparetic leg, which exhibited lower adaptive-based changes (i.e., lower β_adapt_). We speculate that β_adapt_ was lower in patients than in control subjects because it was underestimated due to asymmetry in corrective responses poststroke (Marigold and Eng, 2006; De Kam et al., 2018), rather than because poor recalibration in the nonparetic leg. Recall that $\Delta {\text{EMG}}_{\text{on}(-)}$ was not directly measured. Instead, $\Delta {\text{EMG}}_{\text{on}(-)}$ was estimated from $\Delta {\text{EMG}}_{\text{on}(+)}$ in the contralateral leg. Consequently, any asymmetry in muscle activity would lead to a bad predictor of the corrective responses that would result from recalibration (i.e., ${\text{EMG}}_{\text{on}(-)}$) and thereby reduce the possible β_adapt_. In other words, asymmetries in corrective responses poststroke would result in a poor representation of $\Delta {\text{EMG}}_{\text{on}(-)}$ (i.e., adaptive-based regressor), and thereby underestimation of β_adapt_ for the most asymmetric individuals. We performed an asymmetry-matched regression analysis to test the potential confounding effect of asymmetry on our results. While we could only include a limited number of subjects in this analysis (*n* = 7/group), the asymmetry-matched groups showed that the regression factors quantifying the recalibration of corrective responses were indeed influenced by the asymmetry of stroke survivors. Thus, future studies are needed to determine the potential impact of motor asymmetry on sensorimotor recalibration. Interestingly, these asymmetries affected the estimation of β_adapt_ more in the nonparetic than in the paretic leg, indicating that paretic corrective responses, $\Delta {\text{EMG}}_{\text{on}(+)}$, are a poorer estimate of $\Delta {\text{EMG}}_{\text{on}(-)}$ in the nonparetic leg than vice versa. This is possibly because the missing paretic responses (De Kam et al., 2018) cannot be scaled up to reproduced nonparetic responses, while nonparetic activity can be scaled down to reproduce paretic missing activity. In sum, group differences of the full groups’ nonparetic versus control legs are likely due to underestimation of β_adapt_, rather than to poor sensorimotor recalibration in the nonparetic leg. However, future studies recording $\Delta {\text{EMG}}_{\text{on}(-)}$ are needed to determine whether motor asymmetry is a factor reducing the recalibration of nonparetic corrective responses.

Discrepancies between the paretic and nonparetic extent of adaptive-based changes may reflect leg-specific recalibration. This is supported by the independent recalibration of the legs in hybrid walking (i.e., one leg moving forward faster than the other leg moving backward; Choi and Bastian, 2007). However, leg-specific adaptation in hybrid walking may result from the peculiar nature of this task and may, therefore, not apply to other locomotor adaptation paradigms. In fact, more recent studies have demonstrated interlimb transfer of adapted motor patterns during conventional split-belt walking (Krishnan et al., 2017, 2018), which argues against leg-specific recalibration. Thus, we believe that recalibration of corrective responses is not affected after cerebral lesions, which is also supported by a lack of association between individual stroke survivors’ motor impairments (i.e., Fugl-Meyer scores) and the amount of adaptive-based modulation of corrective responses. Of note, we found an association between β_no-adapt_ and Fugl-Meyer scores driven by stroke survivors with negative β_no-adapt_ values. These negative β_no-adapt_ values, also observed in control subjects (Iturralde and Torres-Oviedo, 2019), may reflect a startle-like (Oude Nijhuis et al., 2010) generic response to an environmental transition, regardless of its direction. Together, our results suggest that, while corrective responses are affected by cerebral lesions (De Kam et al., 2018), their recalibration is not.

### Cerebral lesions affect the execution of updated motor commands in a new walking environment

We found that stroke survivors exhibited impaired modulation of steady-state muscle activity, particularly in their paretic leg. Interestingly, aberrant patterns of muscle activity did not impact the modulation of kinematic asymmetry parameters (Reisman et al., 2007), which may indicate that muscle activity is more sensitive to stroke-related deficits in motor output than parameters quantifying kinematic asymmetries. Previous studies reported that long-term adaptation (i.e., changes in spatiotemporal parameters from baseline walking to late adaptation) is impaired after hemispherectomy (Choi et al., 2009), but not after focal hemisphere lesions due to stroke (Reisman et al., 2007). These observations further suggest that changes in interlimb spatiotemporal parameters become noticeable with more pronounced deficits, whereas more subtle deficits can be detected by analysis of muscle activity. The impaired modulation of muscle activity at steady state in the split-belt walking condition may reflect poor selectivity in the activation of muscles poststroke. Notably, intact individuals upregulated proximal muscles (i.e., quadriceps during early stance and hamstrings during late swing) without increasing distal muscle activity during steady-state split-belt walking. This pattern of modulation was diminished in stroke survivors (Fig. 3), particularly those with poorer leg motor selectivity. This observation is consistent with previous reports of missing selectivity in the activation of proximal and distal muscles in the gait of stroke survivors (Clark et al., 2010). Thus, impaired motor selectivity probably contributes to the aberrant patterns of muscle activity during steady-state split-belt walking. In addition, stroke survivors’ atypical steady-state behavior may also be influenced by their perceptual deficits. Notably, individuals poststroke have difficulty assessing their step-length asymmetry (Wutzke et al., 2015), which could contribute to the adaptation of movements (Hoogkamer et al., 2015a). Alternatively, the atypical muscle activity patterns in stroke survivors may have resulted from a lower walking speed. Indeed, steady-state muscle activity became more similar across groups in our speed-matched analysis. Notably, between-group differences in our similarity metric were still substantial after controlling for speed (0.39 + 0.13 vs 0.28 ± 0.18), but this difference was no longer statistically significant. Therefore, we cannot completely rule out walking speed as a confounding factor influencing the distinct motor patterns at steady state between patients and control subjects. Last, the lack of modulation of steady-state muscle activity was presumably not the result of muscle atrophy, given that muscle groups that lacked modulation in the steady state (e.g., knee extensors) were highly modulated during corrective responses (Fig. 1*D*). We speculate that steady-state muscle activity depends on neural circuits involved in voluntary motor control, whereas this is not the case for corrective responses (De Kam et al., 2018). Together, our results suggest that stroke survivors exhibit impaired execution of updated motor commands in the steady state of split-belt walking, most likely due to their impaired motor function.

### Partial dissociation between recalibration and execution of updated motor commands

We found that stroke survivors exhibited intact recalibration of corrective responses, but impaired muscle patterns at steady-state split-belt walking, suggesting partial dissociation between motor performance in the altered environment and postadaptation behavior. This finding is consistent with previous work demonstrating that the extent to which subjects adapt their movements during split-belt walking does not predict their aftereffects (Sombric et al., 2019). Partial dissociation between steady-state and postadaptation behavior is further supported by the findings that aftereffects are not sensitive to manipulation of steady-state behavior through visual feedback (Wu et al., 2014; Long et al., 2016). Together, our findings suggest that steady-state and postadaptation behaviors are partially independent, and possibly mediated through distinct neural processes.

We found that postadaptation muscle activity was indicative of sensorimotor recalibration of corrective responses also observed in previous studies (Maeda et al., 2018; Iturralde and Torres-Oviedo, 2019). Since recalibration has also been observed in feedforward motor commands on perturbation removal (Tseng et al., 2007; Taylor and Ivry, 2014), our results provide further evidence for shared internal models for generating corrective responses and feedforward motor commands (Wagner and Smith, 2008; Yousif and Diedrichsen, 2012; Cluff and Scott, 2013; Maeda et al., 2018). It has been shown that the cerebellum is involved in feedforward adaptation and learning of internal models. (Martin et al., 1996; Smith and Shadmehr, 2005; Morton and Bastian, 2006). In particular, sensorimotor recalibration in locomotion depends on the intermediate cerebellum (Darmohray et al., 2019), and small focal lesions may not affect it (Hoogkamer et al., 2015b). In addition, the cerebellum may also be involved in the adaptation of corrective responses. This is supported by the cerebellar dependency on timely recruitment (Herzfeld et al., 2014) and the appropriate magnitude of feedback responses to predictable perturbations (Jacobs and Horak, 2007). Together, our results are consistent with the idea that, corrective responses depend on spinal cord and brainstem circuits for their execution (Bolton, 2015; Weiler et al., 2019) and on the cerebellum for their adaptation, which would explain why our participants with cerebral lesions showed intact recalibration of corrective responses.

Our observation of stroke-related impairments in steady-state movement execution suggest that these processes are cerebral dependent, perhaps through connections between cerebral and cerebellar structures (Kelly and Strick, 2003; Hoshi et al., 2005). Moreover, intact motor pathways for voluntary motor control (e.g., corticospinal tract) are most likely involved in the execution of steady-state motor commands (Schweighofer et al., 2018), given our finding that individuals with poorer voluntary motor control also exhibited a more atypical structure of their steady-state muscle activity. Such associations were not found for the execution of corrective responses (De Kam et al., 2018), suggesting that the execution of corrective responses uses different circuitry, most likely at the level of the brainstem (Jacobs and Horak, 2007; Bolton, 2015). Together, our results are consistent with the idea that corrective and planned actions share an internal model, which relies on cerebellar structures for their adaptation and on cerebral structures for their execution.

### Clinical implications

Our detailed characterization of muscle activity modulation during and after split-belt walking allows for the identification of muscle activity that could potentially be targeted by split-belt treadmill training. Our results support previous findings reporting movement aftereffects in stroke survivors comparable to those of control subjects (Reisman et al., 2007, 2013; Lauzière et al., 2016; Lewek et al., 2018). We further show that the extent of sensorimotor recalibration underlying these aftereffects vary greatly across poststroke individuals. We speculate that individual differences in sensorimotor recalibration may explain why some stroke survivors improve their gait symmetry in response to repeated split-belt treadmill training while others do not (Reisman et al., 2013; Betschart et al., 2018; Lewek et al., 2018). If so, it may be possible to identify patients that will benefit from split-belt training within just a single session. Future studies are needed to determine whether individuals’ recalibration of corrective responses can predict their response to repeated training.

## Synthesis

Reviewing Editor: Trevor Drew, University of Montreal Decisions are customarily a result of the Reviewing Editor and the peer reviewers coming together and discussing their recommendations until a consensus is reached. When revisions are invited, a fact-based synthesis statement explaining their decision and outlining what is needed to prepare a revision will be listed below. The following reviewer(s) agreed to reveal their identity: Jacques Duysens. Note: If this manuscript was transferred from JNeurosci and a decision was made to accept the manuscript without peer review, a brief statement to this effect will instead be what is listed below.

The proposed changes (reviewer 1) were introduced a satisfactory manner. However, one issue is still weak. The introduction of 2 hypotheses was added to the intro but the general line still could be improved. Make it clear that these are two opposing hypotheses and then come back to this in the discussion. Something like: The data are not in line with our favorite hypothesis (....) but instead support the hypothesis favoring the cerebellum for recalibration.

Minor corrections:

-“relative to controls, which was surprising given then known deficits in paretic responses post-stroke”... should probably read: .....relative to controls, which was surprising given the

known deficits in paretic responses post-stroke

-440:... startle like... should probably read :........startle-like

-456....These observations further suggest that changes in that interlimb spatiotemporal...... should probably read: ......These observations further suggest that changes in interlimb spatiotemporal

## Author Response

REVIEWER COMMENTS:

Synthesis Statement for Author (Required):

The proposed changes (reviewer 1) were introduced a satisfactory manner. However, one issue is still weak. The introduction of 2 hypotheses was added to the intro but the general line still could be improved. Make it clear that these are two opposing hypotheses and then come back to this in the discussion. Something like: The data are not in line with our favorite hypothesis (....) but instead support the hypothesis favoring the cerebellum for recalibration.

Per the reviewer's request we have updated our summary in the discussion to emphasize that our results did not support our favorite hypothesis, but favored the alternate hypothesize that recalibration of motor commands is not a cerebral-mediated process, but may only depend on intact cerebellar structures.

The updated text reads:

“...Thus, we find an interesting dissociation between execution and recalibration of corrective actions: the execution of motor patterns upon novel demands is cerebral-dependent, but the recalibration is not. These results do not support our original hypothesis that execution and recalibration of corrective responses would be a cerebral-mediated process. Our findings suggest as though this latter process might only depend on other structures such as the cerebellum.”

Minor corrections:

-“relative to controls, which was surprising given then known deficits in paretic responses post-stroke”... should probably read: .....relative to controls, which was surprising given the known deficits in paretic responses post-stroke

We thank the reviewer for pointing this out, this has been changed in the text.

-440:... startle like... should probably read :........startle-like

We thank the reviewer for pointing this out, this has been changed in the text.

-456....These observations further suggest that changes in that interlimb spatiotemporal...... should probably read: ......These observations further suggest that changes in interlimb spatiotemporal

We thank the reviewer for pointing this out, this has been changed in the text.

## References

[B1] Benjamini Y, Hochberg Y (1995) Controlling the false discovery rate: A practical and powerful approach to multiple testing. J R Stat Soc Series B Stat Methodol 57:289–300. 10.1111/j.2517-6161.1995.tb02031.x

[B2] Betschart M, McFadyen BJ, Nadeau S (2018) Repeated split-belt treadmill walking improved gait ability in individuals with chronic stroke: A pilot study. Physiother Theory Pract 34:81–90. 10.1080/09593985.2017.1375055 28901824

[B3] Bhushan N, Shadmehr R (1999) Computational nature of human adaptive control during learning of reaching movements in force fields. Biol Cybern 81:39–60. 10.1007/s004220050543 10434390

[B4] Bolton DAE (2015) The role of the cerebral cortex in postural responses to externally induced perturbations. Neurosci Biobehav Rev 57:142–155. 26321589 10.1016/j.neubiorev.2015.08.014

[B5] Bowden MG, Clark DJ, Kautz SA (2010) Evaluation of abnormal synergy patterns poststroke: Relationship of the Fugl-Meyer assessment to hemiparetic locomotion. Neurorehabil Neural Repair 24:328–337. 10.1177/1545968309343215 19794132 PMC4434590

[B6] Cheung VCK, Turolla A, Agostini M, Silvoni S, Bennis C, Kasi P, Paganoni S, Bonato P, Bizzi E (2012) Muscle synergy patterns as physiological markers of motor cortical damage. Proc Natl Acad Sci U S A 109:14652–14656. 10.1073/pnas.1212056109 22908288 PMC3437897

[B7] Choi JT, Bastian AJ (2007) Adaptation reveals independent control networks for human walking. Nat Neurosci 10:1055–1062. 10.1038/nn1930 17603479

[B8] Choi JT, Vining EPG, Reisman DS, Bastian AJ (2009) Walking flexibility after hemispherectomy: Split-belt treadmill adaptation and feedback control. Brain 132:722–733. 10.1093/brain/awn333 19074191 PMC2664447

[B9] Christensen LO, Andersen JB, Sinkjaer T, Nielsen J (2001) Transcranial magnetic stimulation and stretch reflexes in the tibialis anterior muscle during human walking. J Physiol 531:545–557. 11230526 10.1111/j.1469-7793.2001.0545i.x PMC2278473

[B10] Christensen LOD, Morita H, Petersen N, Nielsen J (1999) Evidence suggesting that a transcortical reflex pathway contributes to cutaneous reflexes in the tibialis anterior muscle during walking in man. Exp Brain Res 124:59–68. 9928790 10.1007/s002210050600

[B11] Clark DJ, Ting LH, Zajac FE, Neptune RR, Kautz SA (2010) Merging of healthy motor modules predicts reduced locomotor performance and muscle coordination complexity post-stroke. J Neurophysiol 103:844–857. 10.1152/jn.00825.2009 20007501 PMC2822696

[B12] Cluff T, Scott SH (2013) Rapid feedback responses correlate with reach adaptation and properties of novel upper limb loads. J Neurosci 33:15903–15914. 10.1523/JNEUROSCI.0263-13.2013 24089496 PMC6618484

[B13] Darmohray DM, Jacobs JR, Marques HG, Carey MR (2019) Spatial and temporal locomotor learning in mouse cerebellum. Neuron 102:217–231.e4. 10.1016/j.neuron.2019.01.038 30795901

[B14] De Kam D, Roelofs JMB, Bruijnes A, Geurts ACH, Weerdesteyn V (2017) The next step in understanding impaired reactive balance control in people with stroke: The role of defective early automatic postural responses. Neurorehabil Neural Repair 31:708–716. 10.1177/1545968317718267 28691582 PMC5714159

[B15] De Kam D, Geurts AC, Weerdesteyn V, Torres-Oviedo G (2018) Direction-specific instability poststroke is associated with deficient motor modules for balance control. Neurorehabil Neural Repair 32:655–666. 10.1177/1545968318783884 29954244

[B16] Finley JM, Long A, Bastian AJ, Torres-Oviedo G (2015) Spatial and temporal control contribute to step length asymmetry during split-belt adaptation and hemiparetic gait. Neurorehabil Neural Repair 29:786–795. 10.1177/1545968314567149 25589580 PMC4501921

[B17] Fugl-Meyer AR, Jääskö L, Leyman I, Olsson S, Steglind S (1975) The post-stroke hemiplegic patient. 1. A method for evaluation of physical performance. Scand J Rehabil Med 7:13–31. 1135616

[B18] Härdle W, Simar L (2007) Applied multivariate statistical analysis. Heidelberg: Springer.

[B19] Herzfeld DJ, Pastor D, Haith AM, Rossetti Y, Shadmehr R, O'Shea J (2014) Contributions of the cerebellum and the motor cortex to acquisition and retention of motor memories. Neuroimage 98:147–158. 10.1016/j.neuroimage.2014.04.076 24816533 PMC4099269

[B20] Hoogkamer W, Bruijn SM, Potocanac Z, Van Calenbergh F, Swinnen SP, Duysens J (2015a) Gait asymmetry during early split-belt walking is related to perception of belt speed difference. J Neurophysiol 114:1705–1712. 10.1152/jn.00937.2014 26203114 PMC4567612

[B21] Hoogkamer W, Bruijn SM, Sunaert S, Swinnen SP, Van Calenbergh F, Duysens J (2015b) Adaptation and aftereffects of split-belt walking in cerebellar lesion patients. J Neurophysiol 114:1693–1704. 10.1152/jn.00936.2014 26203113 PMC4567611

[B22] Hoshi E, Tremblay L, Féger J, Carras PL, Strick PL (2005) The cerebellum communicates with the basal ganglia. Nat Neurosci 8:1491–1493. 10.1038/nn1544 16205719

[B23] Iturralde PA, Torres-Oviedo G (2019) Corrective muscle activity reveals subject-specific sensorimotor recalibration. eNeuro 6:ENEURO.0358-18.2019 10.1523/ENEURO.0358-18.2019 PMC6497908 31043463

[B24] Jacobs JV, Horak FB (2007) Cortical control of postural responses. J Neural Transm (Vienna) 114:1339–1348. 10.1007/s00702-007-0657-0 17393068 PMC4382099

[B25] Jordan MI, Rumelhart DE (1992) Forward models: Supervised learning with a distal teacher. Cogn Sci 16:307–354. 10.1207/s15516709cog1603_1

[B26] Kelly RM, Strick PL (2003) Cerebellar loops with motor cortex and prefrontal cortex of a nonhuman primate. J Neurosci 23:8432–8444. 12968006 10.1523/JNEUROSCI.23-23-08432.2003 PMC6740694

[B27] Kervio G, Carre F, Ville NS (2003) Reliability and intensity of the six-minute walk test in healthy elderly subjects. Med Sci Sports Exerc 35:169–174. 10.1097/00005768-200301000-00025 12544651

[B28] Krishnan C, Ranganathan R, Tetarbe M (2017) Interlimb transfer of motor skill learning during walking: No evidence for asymmetric transfer. Gait Posture 56:24–30. 10.1016/j.gaitpost.2017.04.032 28482202 PMC5499689

[B29] Krishnan C, Washabaugh EP, Reid CE, Althoen MM, Ranganathan R (2018) Learning new gait patterns: Age-related differences in skill acquisition and interlimb transfer. Exp Gerontol 111:45–52. 10.1016/j.exger.2018.07.001 29981399 PMC6119638

[B30] Lauzière S, Miéville C, Betschart M, Duclos C, Aissaoui R, Nadeau S (2016) A more symmetrical gait after split-belt treadmill walking increases the effort in paretic plantar flexors in people post-stroke. J Rehabil Med 48:576–582. 10.2340/16501977-2117 27345026

[B31] Lewek MD, Braun CH, Wutzke C, Giuliani C (2018) The role of movement errors in modifying spatiotemporal gait asymmetry post stroke: A randomized controlled trial. Clin Rehabil 32:161–172. 10.1177/0269215517723056 28750549 PMC5748372

[B32] Long AW, Roemmich RT, Bastian AJ (2016) Blocking trial-by-trial error correction does not interfere with motor learning in human walking. J Neurophysiol 115:2341–2348. 10.1152/jn.00941.2015 26912598 PMC4922458

[B33] Maeda RS, Cluff T, Gribble PL, Pruszynski JA (2018) Feedforward and feedback control share an internal model of the arm’s dynamics. J Neurosci 38:10505–10514. 10.1523/JNEUROSCI.1709-18.2018 30355628 PMC6596259

[B34] Marigold DS, Eng JJ (2006) Altered timing of postural reflexes contributes to falling in persons with chronic stroke. Exp Brain Res 171:459–468. 10.1007/s00221-005-0293-6 16418855 PMC3226801

[B35] Martin TA, Keating JG, Goodkin HP, Bastian AJ, Thach WT (1996) Throwing while looking through prisms: I. Focal olivocerebellar lesions impair adaptation. Brain 119:1183–1198. 10.1093/brain/119.4.1183 8813282

[B36] Merletti R, Parker P (2005) Electromyography. New York: Wiley-Interscience.

[B37] Morton SM, Bastian AJ (2006) Cerebellar contributions to locomotor adaptations during splitbelt treadmill walking. J Neurosci 26:9107–9116. 10.1523/JNEUROSCI.2622-06.2006 16957067 PMC6674518

[B38] Oude Nijhuis LB, Allum JHJ, Valls-Solé J, Overeem S, Bloem BR (2010) First trial postural reactions to unexpected balance disturbances: a comparison with the acoustic startle reaction. J Neurophysiol 104:2704–2712. 10.1152/jn.01080.2009 20810688

[B39] Reisman DS, Wityk R, Silver K, Bastian AJ (2007) Locomotor adaptation on a split-belt treadmill can improve walking symmetry post-stroke. Brain 130:1861–1872. 10.1093/brain/awm035 17405765 PMC2977955

[B40] Reisman DS, McLean H, Keller J, Danks KA, Bastian AJ (2013) Repeated split-belt treadmill training improves poststroke step length asymmetry. Neurorehabil Neural Repair 27:460–468. 10.1177/1545968312474118 23392918 PMC3738184

[B41] Rikli RE, Jones CJ (1998) The reliability and validity of a 6-minute walk test as a measure of physical endurance in older adults. J Aging Phys Act 6:363–375. 10.1123/japa.6.4.363

[B42] Schweighofer N, Wang C, Mottet D, Laffont I, Bakthi K, Reinkensmeyer DJ, Rémy-Néris O (2018) Dissociating motor learning from recovery in exoskeleton training post-stroke. J Neuroeng Rehabil 15:89. 30290806 10.1186/s12984-018-0428-1 PMC6173922

[B43] Smith MA, Shadmehr R (2005) Intact ability to learn internal models of arm dynamics in Huntington’s disease but not cerebellar degeneration. J Neurophysiol 93:2809–2821. 10.1152/jn.00943.2004 15625094

[B44] Sombric CJ, Harker HM, Sparto PJ, Torres-Oviedo G (2017) Explicit action switching interferes with the context-specificity of motor memories in older adults. Front Aging Neurosci 9:1–15. 28321188 10.3389/fnagi.2017.00040 PMC5337495

[B45] Sombric CJ, Calvert JS, Torres-Oviedo G (2019) Large propulsion demands increase locomotor adaptation at the expense of step length symmetry. Front Physiol 10:60. 10.3389/fphys.2019.00060 30800072 PMC6376174

[B46] Taylor JA, Ivry RB (2014) Cerebellar and prefrontal cortex contributions to adaptation, strategies, and reinforcement learning. Prog Brain Res 210:217–253. 10.1016/B978-0-444-63356-9.00009-1 24916295 PMC4118688

[B47] Torres-Oviedo G, Vasudevan E, Malone L, Bastian AJ (2011) Locomotor adaptation. Prog Brain Res 191:65–74. 10.1016/B978-0-444-53752-2.00013-8 21741544 PMC3738197

[B48] Trumbower RD, Finley JM, Shemmell JB, Honeycutt CF, Perreault EJ (2013) Bilateral impairments in task-dependent modulation of the long-latency stretch reflex following stroke. Clin Neurophysiol 124:1373–1380. 10.1016/j.clinph.2013.01.013 23453250 PMC3674210

[B49] Tseng Y-W, Diedrichsen J, Krakauer JW, Shadmehr R, Bastian AJ (2007) Sensory prediction errors drive cerebellum-dependent adaptation of reaching. J Neurophysiol 98:54–62. 10.1152/jn.00266.2007 17507504

[B50] Wagner MJ, Smith MA (2008) Shared internal models for feedforward and feedback control. J Neurosci 28:10663–10673. 10.1523/JNEUROSCI.5479-07.2008 18923042 PMC6671341

[B51] Weiler J, Gribble PL, Pruszynski JA (2019) Spinal stretch reflexes support efficient hand control. Nat Neurosci 22:529–533. 10.1038/s41593-019-0336-0 30742115

[B52] Wolpert DM, Miall RC, Kawato M (1998) Internal models in the cerebellum. Trends Cogn Sci 2:338–347. 10.1016/s1364-6613(98)01221-2 21227230

[B53] Wu HG, Miyamoto YR, Castro LNG, Ölveczky BP, Smith MA (2014) Temporal structure of motor variability is dynamically regulated and predicts motor learning ability. Nat Neurosci 17:312–321. 10.1038/nn.3616 24413700 PMC4442489

[B54] Wutzke CJ, Faldowski RA, Lewek MD (2015) Individuals poststroke do not perceive their spatiotemporal gait asymmetries as abnormal. Phys Ther 95:1244–1253. 10.2522/ptj.20140482 25838335 PMC4556955

[B55] Yousif N, Diedrichsen J (2012) Structural learning in feedforward and feedback control. J Neurophysiol 108:2373–2382. 10.1152/jn.00315.2012 22896725 PMC3545174

